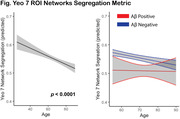# Aging, beta‐amyloid deposition, and brain functional connectivity decline

**DOI:** 10.1002/alz.093945

**Published:** 2025-01-09

**Authors:** Dahyun Yi, Evgeny J. Chumin, Min Soo Byun, Woo‐Jin Cha, Hyejin Ahn, Yu Kyeong Kim, Koung Mi Kang, Chul‐Ho Sohn, Shannon L. Risacher, Olaf Sporns, Kwangsik Nho, Andrew J. Saykin, Dong Young Lee

**Affiliations:** ^1^ Institute of Human Behavioral Medicine, Medical Research Center, Seoul National University, Seoul Korea, Republic of (South); ^2^ Indiana University, Bloomington, IN USA; ^3^ Department of Psychiatry, Seoul National University College of Medicine, Seoul Korea, Republic of (South); ^4^ Department of Neuropsychiatry, Seoul National University Hospital, Seoul Korea, Republic of (South); ^5^ Interdisciplinary program of cognitive science, Seoul National University, Seoul Korea, Republic of (South); ^6^ Department of Nuclear Medicine, SMG‐SNU Boramae Medical Center, Seoul Korea, Republic of (South); ^7^ Department of Radiology, Seoul National University Hospital, Seoul Korea, Republic of (South); ^8^ Center for Neuroimaging, Department of Radiology and Imaging Sciences, Indiana University School of Medicine, Indianapolis, IN USA; ^9^ Department of Psychological and Brain Sciences, Indiana University, Bloomington, IN USA; ^10^ Indiana Alzheimer's Disease Research Center, Indianapolis, IN USA

## Abstract

**Background:**

Changes in brain network organization are influenced by aging. Accumulation of amyloid‐beta (Aß) and neurodegeneration in the neocortex are also expected to alter neuronal networks. Therefore, we examined the relationship between aging and brain functional connectivity (FC), as well as the effect of brain Aß on this relationship.

**Method:**

Resting state functional MRI (rsfMRI) from 594 participants spanning age and diagnostic severity of AD from the Korean Brain Aging Study for the Early Diagnosis and Prediction of AD (KBASE) was preprocessed as previously described in studies conducted at the Indiana AD Research Center (Chumin 2021, 2023). Cortical FC data from 200 regions (Schaefer 2018) grouped into 7 canonical resting state networks (RSN; Yeo 2011) was used to compute a network segregation measure (ratio of within‐ to between‐network connectivity (Chan 2014); here used as an index of FC) across all RSNs. Additionally, a subsample of older participants was classified as Aß positive or negative based on global amyloid in Centiloid units (Klunk 2015).

**Result:**

Intrinsic network connectivity was reduced with increasing age beginning in young adulthood (Fig, left), resulting in a dedifferentiated, or less segregated, network architecture (t = ‐4.79, p = 0.000002). The relationship between age and network segregation was significant in the Aß negative group (t = ‐4.09, p = 0.00005); however, such relationship was not found in the Aß positive group (Fig, right). Fitted Aß values were significantly different (Welch Two Sample t‐test: p < 2.2e‐16).

**Conclusion:**

This preliminary study elucidates age‐related decline of brain FC, quantified as network segregation, from young adulthood to late‐life, wherein RSN communication become less coherent, manifesting as a degeneration of FC structure. Such age‐related reduction pattern of brain connectivity appears disappear under the presence of pathological Aß deposition in brain.